# Supplementary material for: Double-Pulse Femtosecond Laser Fabrication of Highly Ordered Periodic Structures on Au Thin Films Enabling Low-Cost Plasmonic Applications
Source: ACS Nano. 2025 Jun 16;19(25):23258–75. doi: 10.1021/acsnano.5c06177 (PMC12224304; doi:10.1021/acsnano.5c06177)
Supplement: Supplementary file 1 [file nn5c06177_si_001.pdf]

Supplementary Material for

Double-pulse femtosecond laser fabrication of highly-ordered periodic structures on Au thin films enabling low-cost plasmonic applications

Fotis Fraggelakis[1,*], Panagiotis Lingos[1] , George D. Tsibidis[1] , Emma Cusworth[2] , Nicholas Kay[2], Laura Fumagalli[2], Vasyl G. Kravets[2] , Alexander N. Grigorenko[2] , Andrei V. Kabashin[3] and Emmanuel Stratakis[1,4*]

[1]Institute of Electronic Structure and Laser (IESL), Foundation for Research and Technology (FORTH), N. Plastira 100, Vassilika Vouton, 70013 Heraklion, Crete, Greece

[2]Department of Physics and Astronomy, Manchester University, Manchester M13 9PL, UK

[3]Aix Marseille Univ, CNRS, LP3, Campus de Luminy, Case 917, 13288, Marseille, France

[4]Department of Physics, University of Crete, 71003 Heraklion, Crete, Greece

*Correspondence: Fotis Fraggelakis: +302811391940, fraggelakis@iesl.forth.gr, Emmanuel Stratakis: +302810391274, stratak@iesl.forth.gr

1. Electromagnetic Simulations

The two SPP periodicities predicted in this work are derived via the solution of the Maxwell equations at the interfaces of a three-layer (dielectric/metal/dielectric) system and determines the spatial field profile and dispersion of propagating waves for guided electromagnetic modes in waveguides. If the metallic medium is thin enough compared to the penetration depth, such a system is capable of supporting coupled Surface Plasmon Polariton (SPP) modes whose properties can be controlled by the thickness of the metallic film. This can be clearly derived in the dispersion relation of flat thin films in an asymmetric dielectric environment [1,2]

$$exp(-2k_m d) = \frac{k_m/\varepsilon_m + k_a/\varepsilon_a}{k_m/\varepsilon_m - k_a/\varepsilon_a} \cdot \frac{k_m/\varepsilon_m + k_g/\varepsilon_g}{k_m/\varepsilon_m - k_g/\varepsilon_g} \qquad (1)$$

where

$$k_j = \pm\sqrt{\beta^2 - \varepsilon_j k_0^2} \qquad (2)$$

In Eq.1, $j = a, m$ or g denotes the three media (a for air, g for glass, m for metal), d is the thickness of the film, β is the propagation constant of the SPP, and $k_0 = 2\pi/\lambda_L$ is the free-space wavenumber at the laser wavelength. The numerical solution of Eq.1 provides the supported SPP wavelength $\Lambda = 2\pi/Re(\beta)$ for a given film thickness. The calculated wavelengths of the two-interface surface plasmons are dependent on the film thickness. Each interface can sustain bound SPPs. With increasing the film thickness to $d > 35$ nm, the SPPs at the two interfaces become decoupled and separated while strong coupling occurs as the thickness decreases below $\sim$30 nm, while the SPP periodicity decreases abruptly from 687 nm to 395 nm at $d = 5$ nm for the lower SPP mode.

To explore the features of the excited surface waves (which are the precursors of both the periodicity value and the orientation of LIPSS) on an initially non-flat surface, the roughness on Au is emulated with a random distribution of a number of semispherical *bumps* of radius *R*=16 nm (see also Ref.[2]; other relevant work is presented in Ref.[3]). Based on the electromagnetic simulations, the origin of the two observed LIPSS periods are attributed to the SPP periods, excited at the Au-substrate and air-Au interfaces, respectively. More specifically, far- and near- field excited modes are excited (shown in the main manuscript). A Fast Fourier Transform (FFT) analysis of was also shown in the k_x /k_0($\approx \lambda/\Lambda$) space (λ, Λ correspond to the laser wavelength and the periodicity of the modes, respectively) that yield the dual periodicity observed, also, in the experiments. As explained in a previous report [2], bumps scatterers were selected instead of holes in the current work, since in ultra-thin films a hole would lead to direct coupling with the bottom interface. On the other hand, smaller holes would result in similar distributions but with a less efficient excitation of the SPPs (less absorption). Larger nanobumps would lead to multipolar localized modes. Despite the limitations and the profound problematic effects if nanoholes are included, we have made some simulations in which a random distribution of *holes* of radius *R*=15 nm was considered. The electromagnetic profile and the produced near- and far-fields are illustrated in the transverse plane (Figure SM 1a) while the distribution of the electric field in air, metal and dielectric material on the propagation plane are shown in (Figure SM 1b). For $d = 32$ nm the SPP periodicity is $\Lambda = 700$ nm and $\Lambda = 1000$ nm (Figure SM 1c, *green* dot lines) which are very close to the captured periodicity of the energy absorption patterns found by simulation. Both theoretical values are quite close to the experimentally observed periods validating their electromagnetic origin. A precise evaluation of the electromagnetic effects is performed through the employment of Finite Integration Technique (FIT) algorithms to solve Maxwell's equations using the commercial software CST Studio Suite while a brief description of the application of the method assuming scattering of the incident beam of rough surface is presented in Ref.[4].

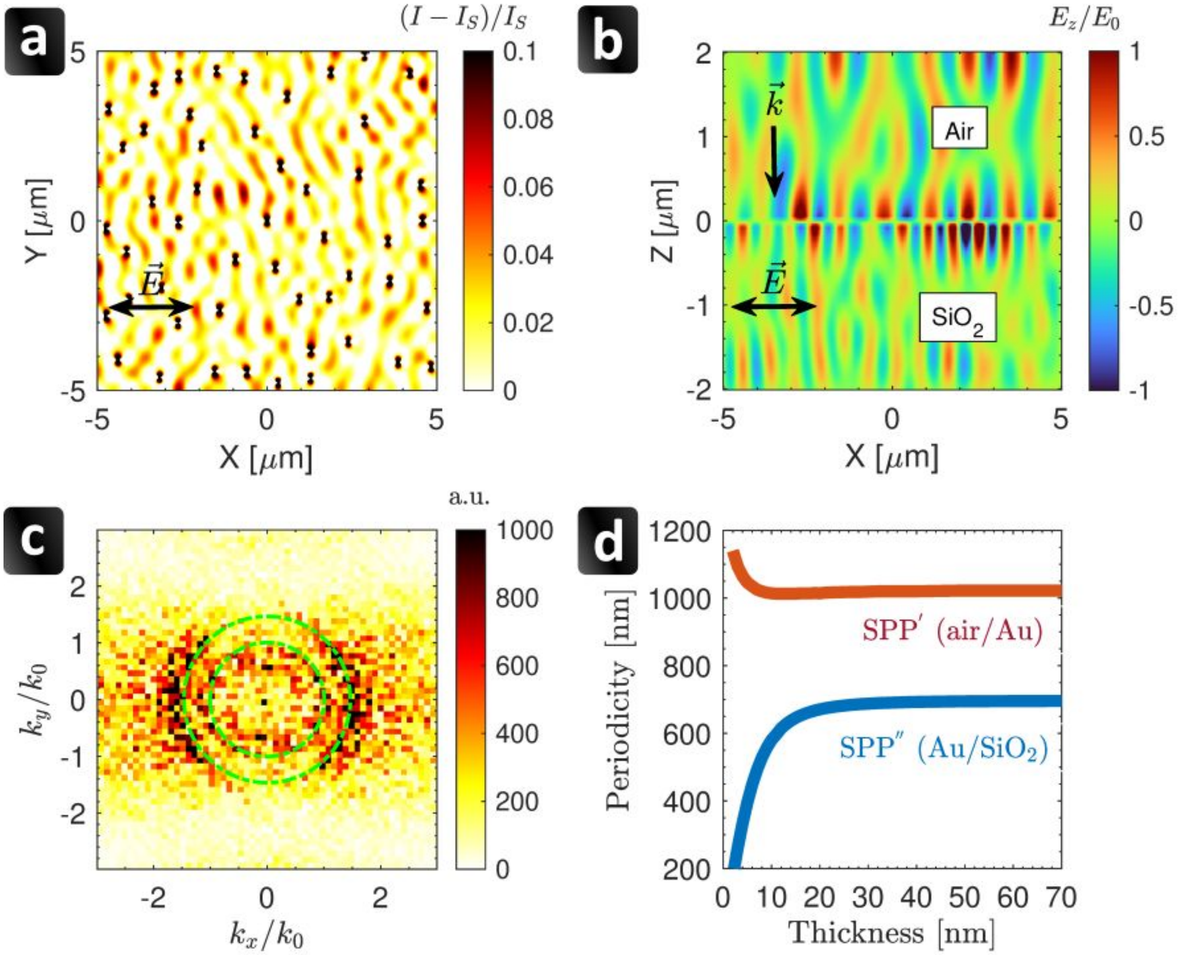

Figure SM 1: (a) The electromagnetic profile and the produced near- and far-fields are illustrated in the transverse plane, (b) distribution of the electric field in air, metal and dielectric material on the propagation plane, (c) Periodicities of the electromagnetic waves, (d) Periodicities of SPP at air/Au and Au/SiO2 interfaces as a function of the Au film thickness.

The numerical solution of Eqs.1-2 provides the supported SPP wavelength $\Lambda = 2\pi/Re(\beta)$ for a given film thickness. The calculated wavelengths of the two-interface surface plasmons are dependent on the film thickness, as illustrated in Figure SM 1. Each interface can sustain bound SPPs.

As it has been also emphasized in a previous work from our group [2], for Au thicknesses larger than 35 nm the long period SPP dominates. By contrast, for very small thicknesses, coupling of the field with the bottom interface becomes stronger and the short SPP wave becomes prominent. For intermediate values of the thickness (including a thickness of ~32 nm), a presence of both SPP is expected. Furthermore, another interpretation can be due to a more realistic representation of an initially rough surface (i.e. emulation of the rough surface with a population of bumps and holes). While in the main manuscript, the electromagnetic fingerprint of bumps was considered, an analysis of the impact of SP excitation due to the presence of holes (presented above) predict the impact of the SPP excited on the lower interface. In particular, a hemispherical hole lead to a direct coupling with the bottom interface which allows a pronounced impact of the electromagnetic mode excited on the Au/SiO_2.

2. Multiscale model and Surface modification

To describe the multiscale process towards surface pattern formation upon irradiating an Au film/substrate two-layered material with femtosecond laser pulses, a theoretical model is used [6, 7, 8]; this model couples together modules that account for the following physical processes occurring at different temporal scales: (i) energy deposition and absorption, (ii) electron excitation and relaxation processes, (iii) phase Transitions (i.e.melting) and ablation, (iv) resolidification.

A detailed employment of the model in case of a two-layered material comprising of a thin film of Au placed on SiO_2 has been presented in a recent report [2]. The inclusion of the pulse separation in the intensity profile is via the use of the expression $I_{total}(x,y,z,t) \sim \left[I(x,y,z) e^{-4\log 2 \left(\frac{t-3\tau_p}{\tau_p}\right)^2} + I'(x,y,z) e^{-4\log 2 \left(\frac{t-3\tau_p-\Delta\tau}{\tau_p}\right)^2} \right]$where τ_p stands for the pulse duration ($\tau_p = 170\ fs$) and $\Delta\tau$ corresponds to the delay between the two subsequent pulses of the double pulse. On the other hand, $I(x,y,z)$ and $I'(x,y,z)$ correspond to the spatial distribution of the intensity for each constituent pulse calculated from the above electromagnetic simulations following a projection of the energy distribution appropriately (so that each constituent pulse has half of the total energy in the double pulse irradiation). For a single pulse, the expression giving $I_{total}(x,y,z,t)$ is $\sim I(x,y,z) e^{-4\log 2 \left(\frac{t-3\tau_p}{\tau_p}\right)^2}$.

The excitation and thermal effects following irradiation of the Au/SiO_2 two-layered material in various conditions is described via the use of a Two Temperature model (TTM) (see a detailed description of the model in Ref. [2,5,8]). It is noted that the onset of criterion of ablation taken in this work considers that it occurs when the lattice temperature exceeds $0.9T_c$=5625 K [5] where T_c (=6250 K) stands for the critical point of Au.

The surface modification is described through the investigation of the dynamics of hydrothermal waves that are developed as a result of the phase transition if the lattice temperature exceeds the melting point [2,5,6]. The laser conditions used in the simulations (i.e. peak fluence equal to $\Phi = 0.28$ J/cm^2) are appropriately selected to ensure the formation of a sufficiently large volume of molten Au after the exposure to the first pulse ($\Phi/2 = 0.14$ J/cm^2). The development and evolution of hydrothermal waves and the dynamics of the produced fluid movement as a result for the phase transformation is mathematically described by the Navier-Stokes Equation (NSE)

$$\rho_0\left(\frac{\partial\vec{u}}{\partial t} + \vec{u}\cdot\vec{\nabla}\vec{u}\right) = \vec{\nabla}\cdot\left(-P + \mu(\vec{\nabla}\vec{u}) + \mu(\vec{\nabla}\vec{u})^T\right) \qquad (3)$$

where μ and ρ_0 stand for the viscosity and density, respectively, of the molten (uncompressed) material, while *P* and $\vec{u}$ are the pressure and velocity of the fluid and superscript *T* denotes the transpose of the vector $\vec{\nabla}\vec{u}$. The solution of NSE is conducted through the employment of appropriate thermocapillary boundary conditions

$$\frac{\partial u}{\partial Z} = -\sigma/\mu \frac{\partial T_L}{\partial X} \text{ and } \frac{\partial v}{\partial Z} = -\sigma/\mu \frac{\partial T_L}{\partial Y} \qquad (4)$$

at the liquid free surface where (*u*,*v*,*w*) are the components of $\vec{u}$ in Cartesian coordinates. In Eq.4, σ stands for the surface tension of the material (see [5,6] for parameter values and for a more detailed description of the fluid dynamics module). As discussed in previous reports [2,5,6,7], inhomogenous energy deposition leads to the development of Marangoni effects (i.e. surface tension-driven molten material flow) and displacement of material from regions of high to low temperatures.

As explained in the main manuscript, the first pulse will lead to a molten profile which will experience the second pulse. Thus, the employment of Eq.3 in addition to the application of the thermal model (lattice temperature profile on the surface of Au is illustrated in Figure SM 2a) will lead to a molten profile at *t*=2 ns which has the height distribution of the hydrothermal wave illustrated in Figure SM 2b.

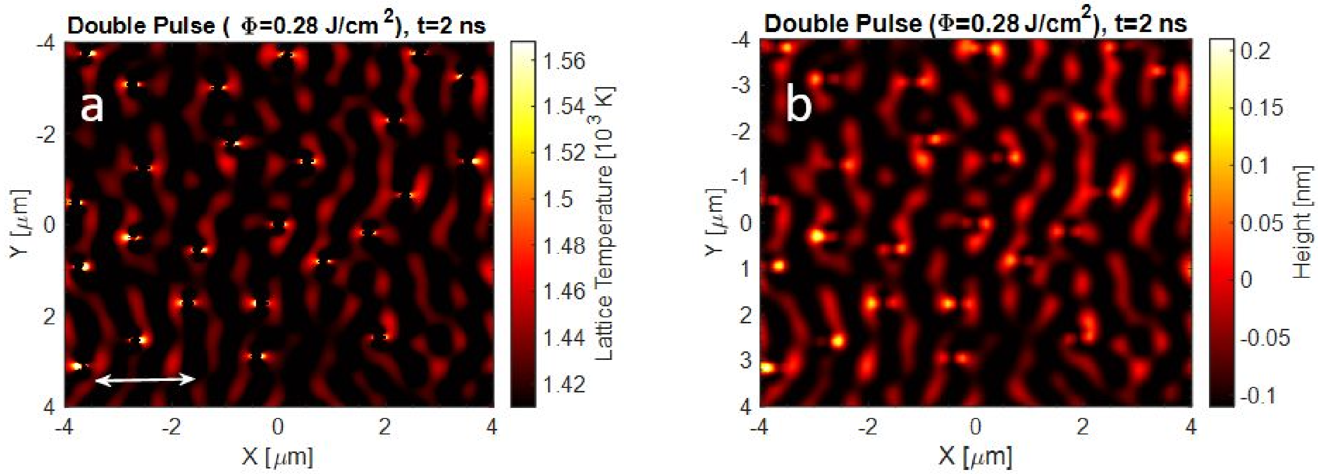

Figure SM 2: (a) Lattice Temperature spatial profile on the surface of Au at *t*=2 ns and (b) Height distribution of molten profile on the surface of Au *t*=2 ns.

3. EDX measurement

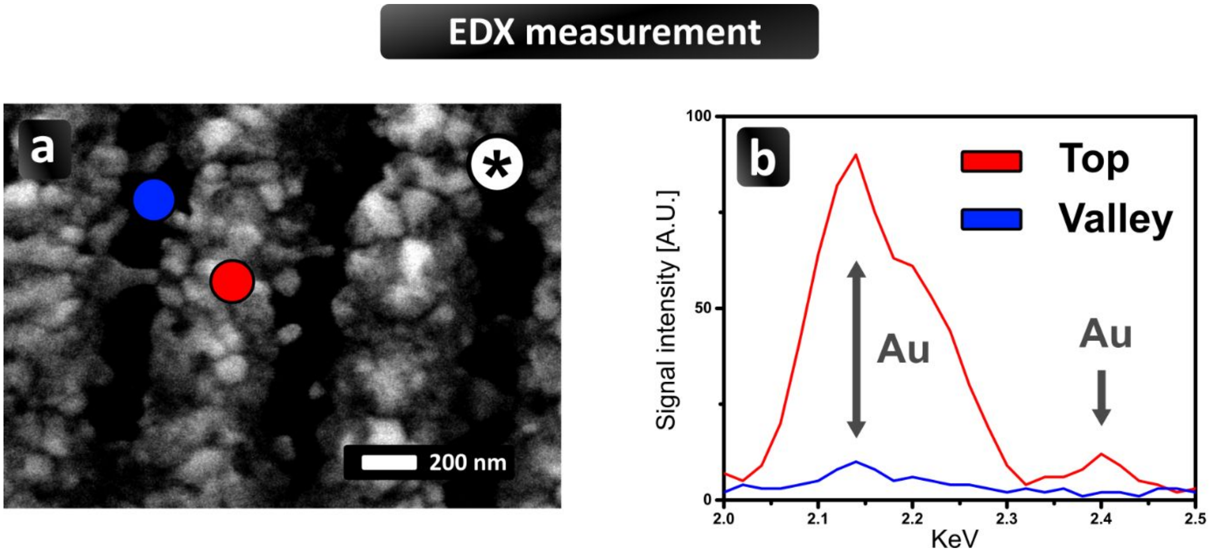

Figure SM 3:EDX measurement. a) SEM image of the examined surface irradiated with Δτ = 1 ns Ov = 300 pps and Φ= 160 mJ/cm^2 corresponding to manuscripts Figure 3ii-DPS. b) Graph showing the signal intensity of EDX measurement versus the eV value for position in valley (blue) and top of ripple structure (read). Characteristic eV values of Au are indicated with arrows.

An EDX measurement was employed to characterize the composition of valleys and ridges for the surface morphology obtained upon irradiation with with *Δτ* = 1 ns *Ov* = 300 pps and *Φ*= 160 mJ/cm^2 annotated with “*’’ in manuscripts Figure 3ii-DPS. The SEM image is shown in Figure SM 3a where the examined areas are marked with in *blue* (valley) and *red* (ridge). The graph in Figure SM 3b shows the EDX graph for varying eV values for the blue and red areas of Figure SM 3a. The characteristic peaks corresponding to Au signal are indicated with black arrows. The sharp contrast between the *red* and *blue* curve confirms the almost complete removal of Au in the areas between LIPSS.

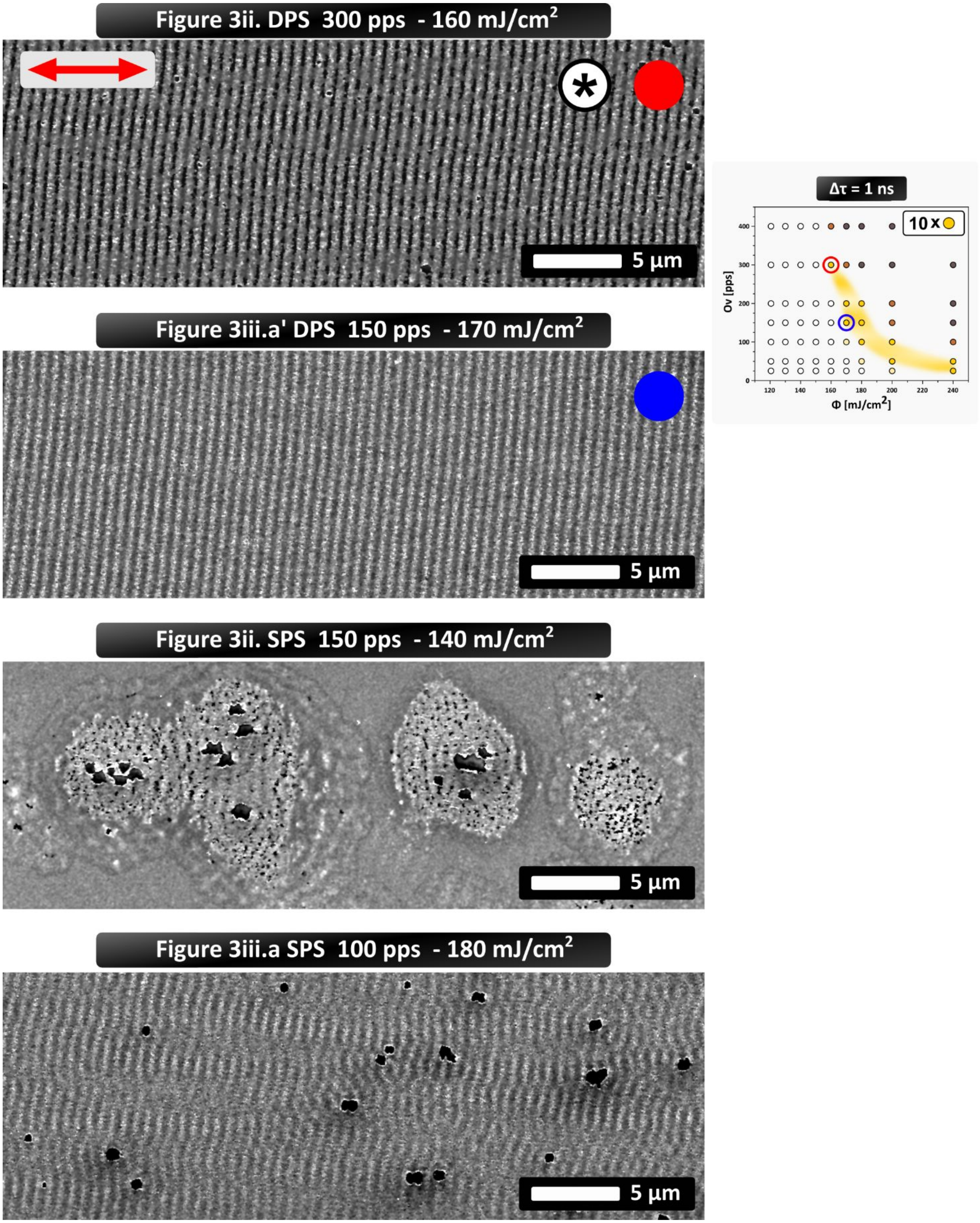

Figure SM 4: High magnification images corresponding to manuscripts Figure 3. Process parameters are indicated. Red arrow indicates the polarization direction

References

[1] Maier, S. A. *Plasmonics: Fundamentals and Applications*; 2007. https://doi.org/10.1007/0-387-37825-1.

[2] P. Lingos, G. Perrakis, O. Tsilipakos, G.D. Tsibidis, Stratakis E., Impact of plasmonic modes on the formation of self organised nano-patterns in thin films, Optics and Laser Technology 163, 109415 (2023).

[3] Rudenko, A.; Mauclair, C.; Garrelie, F.; Stoian, R.; Colombier, J. P. Light Absorption by Surface Nanoholes and Nanobumps. Appl Surf Sci 470, 228–233 (2019).

[4] S. Maragkaki, P. Lingos, G.D. Tsibidis, G. Deligeorgis, E. Stratakis, 'Impact of pre-patterned structures on features of Laser Induced Periodic Surface Structures', Molecules 26 (3) 7330 (2021).

[5] G. D. Tsibidis, M. Barberoglou, P. A. Loukakos, E. Stratakis, C. Fotakis, Dynamics of ripple formation on silicon surfaces by ultrashort laser pulses in subablation conditions, Phys. Rev. B 86 115316 (2012).

[6] F. Fraggelakis, G. D. Tsibidis, E. Stratakis, Ultrashort pulsed laser induced complex surface structures generated by tailoring the melt hydrodynamics, Opto-Electron. Adv. 5 (3) 210052 (2022).

[7] A. Rudenko, C. Mauclair, F. Garrelie, R. Stoian, J.-P. Colombier, Self-organization of surfaces on the nanoscale by topography-mediated selection of quasi-cylindrical and plasmonic waves, Nanophotonics 8 (3) 459–465 (2019).

[8] G.D. Tsibidis, E. Mansour, E. Stratakis, Damage threshold evaluation of thin metallic films exposed to femtosecond laser pulses: the role of material thickness, Optics and Laser Technology 156,108484 (2022).
